# Supplementary material for: Splice-Junction-Based Mapping of Alternative Isoforms in the Human Proteome
Source: Cell Rep. Author manuscript; Available in PMC 2020 Jan 15. (PMC6961840; doi:10.1016/j.celrep.2019.11.026)
Supplement: 2 [file NIHMS1546469-supplement-2.zip › DF1/PXD009737/TestisTrypsin/TestisTrypsin_60_PCBP2_YSTGSDSASFPHTTPSMCLNPDLEGPPLELTK.pdf]

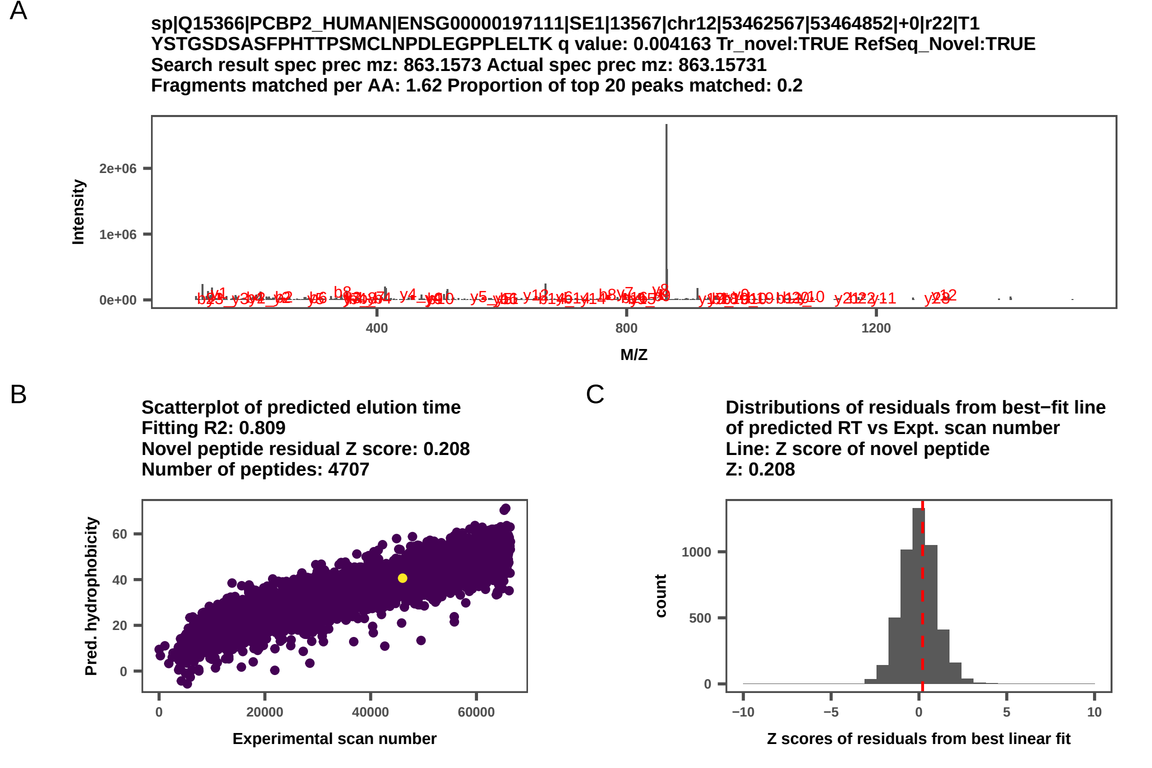
A
sp|Q15366|PCBP2_HUMAN|ENSG00000197111|SE1|13567|chr12|53462567|53464852|+0|r22|T1
YSTGSDSASFPHTTPSMCLNPDLEGPPLELTK q value: 0.004163 Tr_novel:TRUE RefSeq_Novel:TRUE
Search result spec prec mz: 863.1573 Actual spec prec mz: 863.15731
Fragments matched per AA: 1.62 Proportion of top 20 peaks matched: 0.2
Intensity
2e+06
1e+06
0e+00
400
800
1200
M/Z
B
Scatterplot of predicted elution time
Fitting R2: 0.809
Novel peptide residual Z score: 0.208
Number of peptides: 4707
Pred. hydrophobicity
60
40
20
0
0
20000
40000
60000
Experimental scan number
C
Distributions of residuals from best−fit line
of predicted RT vs Expt. scan number
Line: Z score of novel peptide
Z: 0.208
count
1000
500
0
−10
−5
0
5
10
Z scores of residuals from best linear fit
